# Mediation analysis of time‐to‐event endpoints accounting for repeatedly measured mediators subject to time‐varying confounding

**DOI:** 10.1002/sim.8336

**Published:** 2019-08-14

**Authors:** Stijn Vansteelandt, Martin Linder, Sjouke Vandenberghe, Johan Steen, Jesper Madsen

**Affiliations:** ^1^ Department of Applied Mathematics, Computer Science and Statistics Ghent University Ghent Belgium; ^2^ Department of Medical Statistics London School of Hygiene and Tropical Medicine London UK; ^3^ Novo Nordisk Bagsværd Denmark; ^4^ Ghent University Hospital Ghent Belgium

**Keywords:** g‐formula, longitudinal data, mediation, path‐specific effect, time‐dependent confounding

## Abstract

In this article, we will present statistical methods to assess to what extent the effect of a randomised treatment (versus control) on a time‐to‐event endpoint might be explained by the effect of treatment on a mediator of interest, a variable that is measured longitudinally at planned visits throughout the trial. In particular, we will show how to identify and infer the path‐specific effect of treatment on the event time via the repeatedly measured mediator levels. The considered proposal addresses complications due to patients dying before the mediator is assessed, due to the mediator being repeatedly measured, and due to posttreatment confounding of the effect of the mediator by other mediators. We illustrate the method by an application to data from the LEADER cardiovascular outcomes trial.

## 1 INTRODUCTION

There is a growing interest in statistical analyses that support insight into causal mechanisms whereby an exposure affects intermediate variables (or mediators), to then in turn produce an outcome. In many such studies, interest lies in one or more mediators that are measured multiple times during the course of the study. The LEADER trial,^1^ for instance, evaluated the effect of liraglutide (as opposed to placebo), over and above standard care, on time from randomisation to first major adverse cardiovascular event (MACE) in patients with type II diabetes and high cardiovascular risk, in accordance with FDA guidelines. Liraglutide is a once‐daily injectable drug for the treatment of type II diabetes, commonly branded as Victoza. A total of 9340 patients were randomised to either of the two treatments with a median follow‐up time of 3.8 years. The primary analysis showed a protective effect of randomised assignment to liraglutide on time to first MACE, amounting to a hazard ratio of 0.87 (95% confidence interval 0.78 to 0.97; this is based on an intention‐to‐treat analysis, thus not adjusting for time periods off drug, which constituted only a minor fraction of the total patient observation time). Effects were also seen on other endpoints, eg, glycated haemoglobin levels (HbA1c), blood pressure, body weight, and urinary albumin to creatinine ratio, which were all repeatedly assessed at fixed time points 3 to 12 months apart. The question that motivated this research was to what extent the treatment effect on cardiovascular events is mediated by these repeatedly measured intermediate variables.

The literature on structural equation models provides methods for mediation analysis that can handle multiple, repeatedly measured mediators.^2^ The assumptions are strong, however. Besides relying on linear models without interactions for all involved outcomes and mediators, untestable additivity assumptions are often made at the individual level. These assumptions, which are frequently left implicit, state for instance that the effect of treatment while holding the mediators fixed is the same for all individuals, which is biologically implausible.^3^ The causal inference literature on mediation analysis, pioneered by Robins and Greenland^3^ and Pearl,^4^ has explicated these weaknesses and tried to overcome them by providing a framework that is also suitable to nonlinear modelling. However, this framework has mostly confined itself to applications involving single mediators assessed at a single time. The reason is that difficulties of identification tend to pop up as confounders of the mediator‐outcome association are themselves affected by treatment. This happens in particular when multiple mediators are at play or repeatedly measured mediators are assessed, for then the association between (a given) mediator (at a given time) and outcome may be confounded by other or previously measured assessments of the mediator.

Mediation analyses are ideally based on repeated assessments of the mediator for each individual. This is because the scientific interest typically lies in the effect of an exposure mediated via an entire mediator “process.” For example, the interest in the LEADER trial lies in the effect mediated, regardless of the time at which the mediator of interest was assessed. The indirect effect via a single assessment of the mediator is likely to capture only part of the indirect effect via the mediator “process,” as it does not pick up the indirect effects through earlier or later instances of the mediator. The absence of repeated mediator assessments therefore likely results in attenuation of the indirect effect.

When repeatedly measured assessments of the mediator are available, it is tempting to consider simplifying the problem of mediation analysis by aggregating the longitudinal mediators to a single one (eg, in terms of some area under the curve, or the last recorded glycated haemoglobin level). Such simplified mediation analyses may provide a useful starting point but are difficult to justify as the final analysis for various reasons. As previously suggested, a single summary cannot usually capture the full complexity of the mediator, and this may lead to a weakening of the indirect effect. Such simplified mediation analysis moreover prohibits adequate control for confounding. For instance, the area under the curve measurements combine the repeated assessments of the mediator over time; this prohibits adequate control for confounding because some of the covariate measurements measured during the study will then be both the cause and effect of the obtained area under the curve, which typically has undesirable consequences.^5^ Basing the analysis on the last recorded level of the mediator may appear to overcome this problem but is equally problematic. The reason is that the association between the last recorded level of the mediator and the time‐to‐event endpoint is confounded by previously recorded levels of the mediator; adjusting for these would be undesirable as it would eliminate part of the indirect treatment effect. Furthermore, the last recorded level of the mediator may be influenced by the event time itself (whenever the mediator is subject to a period effect), thereby inducing problems of reverse causality. In view of this, we will focus on approaches that explicitly acknowledge the repeated measures nature of the mediator.

As previously suggested, the fact that the association between (a given) mediator (at a given time) and outcome may be confounded by previously measured assessments of the mediator typically complicates identification. The effect of exposure on outcome as transmitted along a single pathway (eg, the effect via the first assessment of the mediator alone) is however generally not of interest when the mediator is repeatedly measured: the primary scientific interest then lies in the effect via the “mediator process,” which is defined by a combination of pathways that involve the different assessments of the mediator over time. Interestingly, closer examination of the identification results for mediation analysis clarifies that the effect of exposure on outcome as transmitted along specific combinations of pathways (eg, involving different or repeatedly measured mediators) is sometimes easier to identify.^6^ In this article, we will therefore make use of a general theory of identification in nonparametric structural equations models with independent errors and possible latent variables^6^ to infer the effect of exposure on outcome transmitted along a combination of pathways. In doing so, we will address complications of working with a time‐to‐event endpoint, such as that individuals may die before the mediator is assessed.

In the next section, we describe the setting under which we will work. In Section 3, we first discuss our proposal to infer the direct and indirect effect of interest accounting for repeatedly measured mediators subject to time‐varying confounding. We start with explaining how one may calculate these effects and end with a discussion of how the proposed approach can be viewed as a generalisation of dynamic path analysis,^7^ as well as how it relates to alternative proposals.^8^, ^9^, ^10^ In Section 4, we present the method applied to the LEADER data and compare the results to less complex methods (all variants of the common Cox regression model), and in Web Appendix B, we present a simulation study in order to evaluate the behaviour of our proposal. We conclude with some final remarks and ideas about possible extensions in Section 5.

## 2 SETTING

Consider a study design that randomises independent patients *i*=1,…,*n* over two treatment arms *A*_*i*_, coded 1 for treatment and 0 for control, and intends to subsequently record longitudinal measurements of the mediator *M*_*i*1_,…,*M*_*ik*_ at visits 1,…,*k*, along with a time‐to‐event endpoint *T*_*i*_. In actual fact, mediator measurements are only recorded until the end‐of‐study time *k* or until the event of interest happens, whichever comes first. Our results will also be applicable to nonrandomised exposures, as they will accommodate adjustment for possible baseline confounding variables *L*_0_. Furthermore, the time‐to‐event endpoint may be censored administratively or due to loss to follow‐up. We assume, for notational convenience, that all patients are seen at the same, equidistant, time points. The latter restriction is readily relaxed (by using time points *t*_*i*1_,…,*t*_*ik*_), provided that these observation times are noninformative (and the no unmeasured confounding assumptions that we will assume, remain plausible). We moreover assume the absence of competing risks at this point, which we will address in the discussion of this paper.

**Figure 1:**
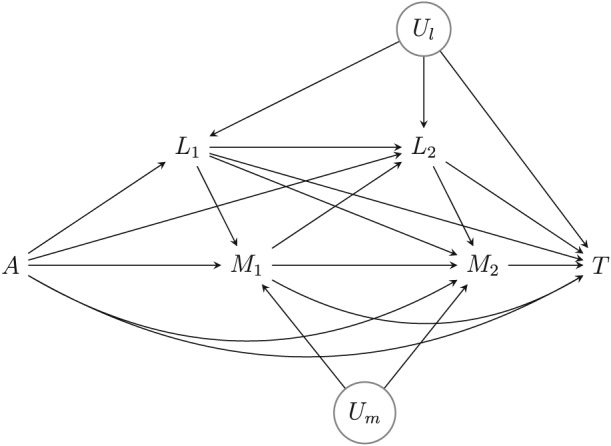
Causal diagram. *U*_*m*_ and *U*_*l*_ refer to unmeasured variables. The measured time‐varying confounders *L*_1_ and *L*_2_ include survival at visits 1 and 2 (*T* may thus be viewed as survival beyond visit 2). Besides the assumptions embodied in this diagram, we assume that censoring at each time is noninformative in each trial arm, given the history of measured time‐varying confounders and mediators at that time, in the sense defined in the main text

Informally, in relation to the example from the LEADER trial, our proposed analysis will infer how different the risk of being event free at a given time would be in the liraglutide arm if the mediator levels for each patient in that arm changed to the levels that we would have seen for that patient on the placebo arm; we will give a formal description in Section 3. Because such mediation analysis conceptualises modifying the mediator, it will be important to control for confounding of the association between mediator and outcome at each time. Our subsequent analysis, like nearly all mediation analyses in the literature, assumes that sufficient data are available on prognostic factors of the event of interest that are also associated with the mediator, to trust that sufficient control for confounding can be made. We will use *L*_*i*0_,*L*_*i*1_,…,*L*_*ik*_ to denote those confounders (eg, concomitant medication, …) measured for patient *i* at baseline and at visits 1,…,*k*, respectively; *L*_*it*_ in particular includes the at‐risk indicator *I*(*T*>*t*), which is 1 for subjects who are event free at time *t* and 0 otherwise. Thus, *L*_*i*0_ refers to baseline covariates (eg, age, gender, baseline level of the mediator, …), and *L*_*i*1_,…,*L*_*ik*_ refer to potential confounders measured at visits 1,…,*k*, the same visits at which the mediator levels were assessed. Importantly, we will assume throughout that those confounders *L*_*it*_ measured at visit *t* are not influenced by the mediator level assessed at that time, although we will allow for those confounders to affect mediator levels at time *t* (as well as at later times) and for them to have been influenced by mediator levels at earlier time points as in Figure 1. When only previous confounder measurements are known to influence the mediator at visit *t* and the mediator at visit *t* may influence confounders at visit *t* and later times, then one must redefine *L*_*it*_ to include only covariates measured at visit *t*−1.

The causal diagram in Figure 1 visualises the data‐generating mechanism that we will postulate, in the absence of censoring (the additional complications posed by censoring of the event time will be addressed later). We will assume throughout that it represents a nonparametric structural equation model with independent errors.^11^, ^12^ It shows a clear, prespecified causal ordering of the confounder and mediators over time, as considered in the works of VanderWeele and Vansteelandt^13^ and Steen et al^14^ for cross‐sectional multiple mediator settings. In spite of this, we will not adopt the proposal by these authors for two reasons. First, it infers the mediated effect via each of the mediators at each time separately, which gives a more refined decomposition than we are aiming for. Indeed, our interest lies in the effect mediated by *M*_*i*1_,…,*M*_*ik*_, regardless of the specific time. In particular, we will infer the effect of randomised assignment to treatment as transmitted along the combination of pathways whereby treatment *directly* influences one of the mediators *M*_*i*1_,…,*M*_*ik*_ (not via *L*_*i*1_,…,*L*_*ik*_), which may in turn influence the risk of the event of interest through an arbitrary mechanism (possibly via *L*_*i*2_,…,*L*_*ik*_). Second, in their sequential approach, VanderWeele and Vansteelandt^13^ and Steen et al^14^ generally assume that the mediators share no unmeasured common causes and moreover assume that none of the intermediate confounders shares unmeasured common causes with the outcome. We will relax these assumptions for two reasons. First, to render the required no unmeasured confounding assumptions plausible, *L*_*i*0_,*L*_*i*1_,…,*L*_*ik*_ will likely include a large number of covariates, some of which may be influenced by treatment. This makes it unlikely that all these components are only associated with the endpoint of interest by means of a causal effect. In our development below, as in Figure 1, we will therefore allow for the association between the covariates *L*_*i*0_,*L*_*i*1_,…,*L*_*ik*_ and the outcome to be itself confounded by possibly unmeasured factors *U*_*l*_. In fact, our proposal will also be valid when—unlike suggested by Figure 1—these unmeasured factors are time varying and influenced by the history, including treatment. Second, it would be unlikely that repeated assessments of the mediator for the same individual have no unmeasured causes in common. In our development below, as in Figure 1, we will therefore allow for the association between the mediators *M*_*i*1_,…,*M*_*ik*_ to be affected by possibly unmeasured factors *U*_*m*_. Traditional longitudinal mediation analyses,^2^ as well as dynamic path analysis that can be viewed as an extension thereof to survival endpoints,^7^, ^15^ implicitly assume the absence of such common causes (or frailties) *U*_*l*_ and *U*_*m*_ because they attempt a more refined decomposition of the exposure effect. They moreover invoke Markov assumptions that assume the absence of long‐term effects of covariates and mediators on covariates and mediators measured later in time; such assumptions are easy to avoid in our proposed approach below.

Note that the mediated effect on which we will focus excludes pathways whereby treatment initially influences time‐dependent patient characteristics *L*, which then in turn influence the mediator and thereby the risk of the event. Those pathways will be attributed to the indirect effect via those patient characteristics. This seems logical from an interpretational point of view, but is also a more fundamental requirement: We will later see that the effect of treatment transmitted along the combination of *all* pathways that intercept one or multiple mediators *M*_*i*1_,…,*M*_*ik*_ (regardless of where in the causal chain it intercepts these variables) cannot be identified without making overly stringent assumptions.

## 3 PROPOSAL

### 3.1 Estimands

To define the direct and indirect effects of interest, we will make use of the so‐called path‐specific effects,^16^ expressed as differences or ratios of survival probabilities. In particular, we will calculate how likely it would be to be event free at a chosen time *t* in the experimental treatment arm if the mediator levels for each patient changed to the levels that we would have seen if that patient had been assigned to control, but the levels of the time‐varying confounders had otherwise remained unchanged. That is, the probability for a randomly chosen patient to be event free at time *t* on treatment if *L*_1_ took on the value *L*_1_(1) that we would have seen for that patient on treatment, if *M*_1_ were set to the level *M*_1_(0,*L*_1_(1)) that we would have seen on control for that patient if *L*_1_ had been set to the previously chosen value, if *L*_2_ were set to the level *L*_2_(1,*M*_1_(0,*L*_1_(1))) that we would have seen on treatment for that patient if *L*_1_ and *M*_1_ had been set to the previously chosen values, and so on (see the Appendix for more details). Let us denote the corresponding probability *S*_1,0_(*t*). When repeating this for all times *t*, we obtain two survival curves, which one may contrast to visualise the targeted path‐specific effect via *M*. In particular, one may express it as the contrast *S*_1,1_(*t*)/*S*_1,0_(*t*), *S*_1,1_(*t*)−*S*_1,0_(*t*), or {1−*S*_1,1_(*t*)}/{1−*S*_1,0_(*t*)} for each time *t*. One may additionally contrast *S*_1,0_(*t*) with how likely it is to be event free at that time in the control arm. Let us denote this probability *S*_0,0_(*t*). If we repeat this for all times *t*, we will once more obtain two survival curves which we may then contrast to visualise the path‐specific effect not via *M*. In particular, one may express this as the contrast *S*_1,0_(*t*)/*S*_0,0_(*t*), *S*_1,0_(*t*)−*S*_0,0_(*t*), or {1−*S*_1,0_(*t*)}/{1−*S*_0,0_(*t*)} for each time *t*. The mediated proportion can then be visualised as the ratio of the path‐specific effect via *M* to the total intention‐to‐treat effect over time:

$$\frac{S_{1,1}(t)-S_{1,0}(t)}{S_{1,1}(t)-S_{0,0}(t)}.$$

In the example from the LEADER trial, one may moreover have interest in contrasting *S*_1,1_(*t*) and *S*_0,0_(*t*) with *S*_0,1_(*t*), the survival probability at time *t* in the control arm if the mediator levels for each patient changed to the levels that we would have seen if that patient had been assigned to experimental treatment, but the levels of the time‐varying confounders had otherwise remained unchanged. The methodology presented in this article can easily be applied to derive this contrast as well.

One subtlety in the interpretation of *S*_1,0_(*t*) is that some patients may die sooner when assigned to control than when assigned to experimental treatment, in which case their mediator values on control may appear ill defined until the considered time *t*.^8^ Note however that the variables *L*_*t*_,*t*=1,…,*k*, include the at‐risk indicator *I*(*T*>*t*). Setting *M*_1_ to the level *M*_1_(0,*L*_1_(1)), *M*_2_ to the level *M*_2_(0,*L*_1_(1),*M*_1_(0,*L*_1_(1)),*L*_2_(1,*M*_1_(0,*L*_1_(1)))), …thus amounts to setting the mediators to the level they would have taken in the control arm if the history of time‐varying confounders, including the survival status at that time, were set to the level on the experimental arm. We will discuss implications in more detail in the discussion section (see also Web Appendix A).

In the forthcoming sections, we will explain how one may calculate the probabilities *S*_1,0_(*t*) for different times *t*. The probabilities *S*_1,1_(*t*) and *S*_0,0_(*t*) can likewise be obtained upon reversing the codings 0 and 1 in the proposal below. They could also be obtained directly from a standard nonparametric analysis of both treatment arms, although we recommend calculating them in a model‐based way as suggested below, to give results that are better comparable with the calculation of *S*_1,0_(*t*). Our results in this section derive from the general identification results in Web Appendix A. These identification results can be obtained via application of the edge g‐formula,^17^ which is a generalisation of the well‐known g‐formula to the identification of the distribution of the so‐called nested counterfactuals (or counterfactual responses to the so‐called edge interventions). The identification results are nonparametric, suggesting that arbitrary models can be used in each of the stages. However, below, we will suggest the use of Cox regression models as a special case.

### 3.2 General procedure

In Web Appendix A, we show formally that the chance $S_{a,a^{*}}(t)$, for given values *a* and *a* ^∗^, can formally be identified as

$$\begin{array}{ll} S_{a,a^{*}}(t)= & \int f\left(T>t|T>\lfloor t\rfloor ,{\bar{m}}_{\left\lfloor t\right\rfloor },{\bar{l}}_{\left\lfloor t\right\rfloor },A=a\right)\\ & \times \overset{\left\lfloor t\right\rfloor }{\underset{s=1}{\prod }}f\left(m_{s}|T>s,{\bar{l}}_{s},{\bar{m}}_{s-1},A=a^{*}\right)f\left(l_{s}|T>s-1,{\bar{l}}_{s-1},{\bar{m}}_{s-1},A=a\right)dm_{s}dl_{s}, \end{array}$$

where ⌊*t*⌋ is the visit time prior to (and including) time *t*, and we define ${\bar{m}}_{s}\equiv (m_{1},\text{…},m_{s})$, ${\bar{l}}_{s}\equiv (l_{1},\text{…},l_{s})$, and *m*_0_=*∅*; here, $f(m_{s}|T>s,{\bar{l}}_{s},{\bar{m}}_{s-1},A=a^{*})$ is shorthand notation for $f(M_{s}=m_{s}|T>s,{\bar{L}}_{s}={\bar{l}}_{s},{\bar{M}}_{s-1}={\bar{m}}_{s-1},A=a^{*})$. Specialising this to times *t* between the first and second mediator assessments and noting that *L*_1_ is composed of the at‐risk indicator *I*(*T*>1) and patient characteristics *V*_1_, the chance *S*_1,0_(*t*) can formally be calculated as

(1) $$\begin{array}{ll} \int & P(T>t|T>1,A=1,m_{1},v_{1},l_{0})f(m_{1}|T>1,A=0,v_{1},l_{0})\\ & \times \;f(v_{1}|T>1,A=1,l_{0})P(T>1|A=1,l_{0})f(l_{0})dm_{1}dv_{1}dl_{0}. \end{array}$$

The above identification results show some similarity to the g‐formula and the mediational g‐formula,^18^ but do not follow from those theories, which apply to nonnested counterfactuals only.

Monte Carlo integration can be used for evaluating the above identity and, moreover, gives it intuitive meaning. This involves first fixing *L*_0_ for each individual *i*=1,…,*n* at the observed value *l*_0*i*_. Next, for each individual, a (possibly counterfactual) event time *t*_*i*_(1) is drawn from the distribution *f*(*t*|*A*=1,*l*_0*i*_), and next for individuals with *t*_*i*_(1)>1, the counterfactual level *V*_1_(1) is fixed at a random draw *v*_1*i*_(1) from the distribution *f*(*v*_1_|*T*>1,*A*=1,*l*_0*i*_). Subsequently, for each individual with *t*_*i*_(1)>1, *M*_1_(0,*L*_1_(1)) is fixed at a random draw *m*_1*i*_(0,*l*_1*i*_(1)) from the distribution *f*(*m*_1_|*T*>1,*A*=0,*V*_1_=*v*_1*i*_(1),*l*_0*i*_), and *T*(1,*L*_1_(1),*M*_1_(0,*L*_1_(1))) is fixed at a random draw *t*_*i*_(1,*l*_1*i*_(1),*m*_1*i*_(0,*l*_1*i*_(1))) from the distribution *f*(*t*|*T*>1,*A*=1,*M*_1_=*m*_1*i*_(0,*l*_1*i*_(1)),*V*_1_=*v*_1*i*_(1),*l*_0*i*_); for each individual with *t*_*i*_(1) ≤ 1, *t*_*i*_(1,*l*_1*i*_(1),*m*_1*i*_(0,*l*_1*i*_(1))) is fixed at *t*_*i*_(1). The chance *S*_1,0_(*t*) can then be estimated as the proportion of individuals with *t*_*i*_(1,*l*_1*i*_(1),*m*_1*i*_(0,*l*_1*i*_(1)))>*t*.

The main drawback of the above Monte Carlo strategy is that it involves modelling the joint distribution of all variables, which becomes especially cumbersome when *L*_1_ is high dimensional. Upon rewriting (1) in terms of a series of nested conditional expectations,

$$E(E[E\{P(T>t\;|\;T>1,A=1,M_{1},L_{1},L_{0})\;|\;T>1,A=0,L_{1},L_{0}\}\;|\;T>1,A=1,L_{0}]\times P(T>1\;|\;A=1,L_{0}))$$

(in line with a common representation of the g‐formula), repeated regressions can be used instead (see Web Appendix A for more detail). We will illustrate this for the data structure in Table 1, which shows artificial data for 10 patients; here, *E*_*i*_ is an indicator for the event (1 if the event occurred while enrolled in the trial, 0 otherwise).

**Table 1:** A toy example for a restricted set of patients

Patient	*A*_*i*_	*L*_0*i*_	*L*_1*i*_	*M*_1*i*_	*E*_*i*_	*T*_*i*_	$Q_{i}^{1}(t)$	$Q_{mi}^{1}(t)$	$Q_{li}^{0}(t)$	$Q_{i}^{0}(t)$
1	0	62.79	62.36	63.36	1	5.57	0.81	0.79	0.82	0.98
2	0	64.75	65.96	75.78	1	0.65	.	.	0.82	0.97
3	0	57.13	56.35	74.80	1	11.44	0.77	0.80	0.83	0.98
4	1	56.28	55.27	53.08	1	9.42	0.84	0.80	0.83	0.98
5	1	72.55	68.05	59.07	1	13.68	0.85	0.82	0.82	0.97
6	1	67.61	61.02	54.17	1	9.61	0.87	0.84	0.82	0.98
7	0	52.84	46.85	65.93	1	6.70	0.84	0.84	0.83	0.98
8	1	65.16	58.16	51.88	0	24.00	0.88	0.84	0.82	0.97
9	0	62.69	59.91	66.51	1	5.61	0.82	0.81	0.82	0.98
10	1	74.23	65.88	50.62	0	24.00	0.89	0.85	0.82	0.97

In particular, at each time *t*, the chance *S*_1,0_(*t*) can be calculated as follows.

1. Fit a Cox regression model among people who survived the previous visit in the experimental arm in function of the history of mediators and confounders up to that visit, accounting for censoring in the default way. This analysis accommodates noninformative censoring, given the history of measured mediators and confounders up to the considered visit. Next, use the fitted model to predict the chance of surviving the given time *t* for each patient in the study who survived the previous visit, setting the mediators and confounders to their observed values. Denote the result $Q_{i}^{\left\lfloor t\right\rfloor }(t)$ for patient *i*. Besides maximum partial likelihood estimators of the regression coefficients, this requires an estimator of the cumulative baseline hazard, for which we used the Breslow estimator. For instance, with parameter estimates ${\hat{\alpha }}_{1}=0.02,{\hat{\alpha }}_{2}=0.05$, and ${\hat{\alpha }}_{3}=-0.05$ for the log hazard ratio corresponding to *M*_1_,*L*_0_, and *L*_1_, respectively, the chance of surviving the given time *t* (eg, 5 months) for the first patient is $\exp[-{\hat{\Lambda }}_{0}(5)\times \exp\{(0.02\;\times \;63.36)+(0.05\;\times \;62.79)+(-0.05\;\times \;62.36)\}]$, with ${\hat{\Lambda }}_{0}(5)$ the estimated cumulative baseline hazard, which equalled 0.06 after 5 months.
2. Next, repeat the following for each of the previous visits *k*=⌊*t*⌋ to 1.
  (a) Regress *Q* ^*k*^(*t*) on the history of the mediators ${\bar{M}}_{k-1}=(M_{1},\text{…},M_{k-1})$ and the history of the covariates ${\bar{L}}_{k}=(L_{0},\text{…},L_{k})$ among people who were event free at visit *k* in the control arm. Because *Q* ^*k*^(*t*) lies between 0 and 1, a quasi‐binomial regression with logit link may be an appropriate choice. Next, we can use the model to calculate a prediction $Q_{m}^{k}(t)$ for all subjects who were event free at visit *k*, based on their observed data on mediators and confounders. For instance, with parameter estimates ${\hat{\beta }}_{0}=1.48,{\hat{\beta }}_{1}=0.049$, and ${\hat{\beta }}_{2}=-0.051$ for the intercept and the log odds ratios corresponding to *L*_0_ and *L*_1_, respectively, the prediction $Q_{mi}^{k}(t)$ for *k*=1 for the first patient who was event free at the first visit would be calculated as expit{1.48+(0.049  × 62.79)+(−0.051  × 62.63)} and would equal 0.79.
  (b) Regress $Q_{m}^{k}(t)$ on the history of mediators ${\bar{M}}_{k-1}$ and confounders ${\bar{L}}_{k-1}$ among people who were event free at visit *k* in the experimental treatment arm. Because $Q_{m}^{k}(t)$ lies between 0 and 1, a quasi‐binomial regression with logit link may be an appropriate choice. Use the model to calculate a prediction $Q_{l}^{k-1}(t)$ for all patients who were event free at visit *k*−1, based on their observed data on mediators and confounders. For instance, with parameter estimates ${\hat{\delta }}_{0}=1.71$ and ${\hat{\delta }}_{1}=-0.003$ for the intercept and the log odds ratio corresponding to *L*_0_, the prediction $Q_{li}^{k-1}(t)$ for *k*=1 for the first patient is expit{1.71+(−0.003  × 62.79)} and equals 0.82. Note that $Q_{l}^{k-1}(t)$ is now also estimated for patients who were not event free at visit *k*, using their data on ${\bar{M}}_{k-1}$ and ${\bar{L}}_{k-1}$.
  (c) Fit a Cox model among people who were event free at visit *k*−1 in the experimental treatment arm, in function of the history ${\bar{M}}_{k-1}$ and ${\bar{L}}_{k-1}$. Use the fitted model to estimate the chance of surviving visit *k* for each patient in the study who was event free at visit *k*−1, setting ${\bar{M}}_{k-1}$ and ${\bar{L}}_{k-1}$ to the observed covariate values. Let *Q* ^*k*−1^(*t*) denote the product of this predicted value and the value of $Q_{l}^{k-1}(t)$ obtained in the previous step. For instance, with the following parameter estimate for the log hazard ratio, ${\hat{\theta }}_{1}=0.003$, the probability of being event free at the first visit on the experimental treatment is calculated as $\exp[-{\hat{\Lambda }}_{0}(3)\times \exp\{0.003\;\times \;62.79\}]$ for patient 1, with ${\hat{\Lambda }}_{0}(3)$ the estimated cumulative baseline hazard, which equalled 0.02 after the first visit.
3. When the previous steps have been repeated for visits *k*=⌊*t*⌋ to 1, then average the value *Q* ^0^(*t*) obtained in the final step across all patients. The resulting average is an estimate of *S*_1,0_(*t*). Averaging *Q* ^0^(*t*) across all patients in the example would thus result in an estimate of *S*_1,0_(*t*) at 5 months, which equals 0.80.

Note that step 2(a) involves regressing predictions among patients who were event free in the control arm (in order to integrate over the distribution of the mediator), whereas step 2(b) involves regressing predictions among patients who were event free in the experimental treatment arm (in order to integrate over the distribution of time‐varying confounders). This differential selection of patients in the respective steps is reflective of the “construction” of nested counterfactuals.

In the above procedure, we have chosen to use separate Cox models at each time at which intermediate variables are assessed. Under correct specification of these models, the required censoring assumption is that censoring at each study visit *k* is noninformative, in the sense that the decision to discontinue the trial at a given time has no residual dependence on the remaining survival time amongst (alive and participating) patients in the same trial arm with the same history of the observed measurements ${\bar{M}}_{k}$ and confounders ${\bar{L}}_{k}$ at that time. Alternatively, one may choose to obtain predictions from a Cox model with time‐varying covariates and avoid the need for separate Cox regression models at each visit time. This approach has the advantage of yielding potentially more precise predictions as this Cox regression model is fitted on the whole sample. The disadvantage however is a greater risk of misspecifying this Cox regression model. In the above procedure, we could alternatively have used parametric survival models using splines for the time effect^19^ or binomial regression models for the chance of surviving time *t*, separately for each time *t*. We have not considered the latter option because the information may become sparse at the later time points, which does not pose complications when relying on the proportional hazards assumption in the Cox regression model.

For computational convenience and to limit the modelling efforts, we have chosen to use binomial regression models to model the predictions obtained from the Cox model. One concern about this strategy is that the considered binomial regression model may fail to be congenial with the chosen Cox model. In view of this, we recommend the use of quasi‐binomial regression with a logit (as opposed to probit) link. The use of such models ensures that when the above procedure is employed for the calculation of *S*_0,0_(*t*) or *S*_1,1_(*t*), misspecification (and in particular lack of congeniality) of the logistic regression models for $Q_{m}^{k}(t)$ and $Q_{l}^{k}(t)$ does not induce bias because the average of the fitted values from a logistic regression model (in the treatment or control arm) equals the average of the outcome under that model (in the treatment or control arm), regardless of whether the model is correctly specified.^20^ To additionally ensure unbiased estimation of *S*_1,0_(*t*) or *S*_0,1_(*t*), we further recommend that these logistic regression models obey the structure of the models that were used to obtain the predictions which they use as input. For instance, if the Cox model contains interactions between *A* and *L*_*k*_, then these should also be included in the logistic regression model for *Q* ^*s*^(*t*),*s*<*k*; if the Cox model contains interactions between *A* and *M*_*k*_, then these will likely give rise to interactions between *A* and predictors of *M*_*k*_ in the logistic regression model for *Q* ^*s*^(*t*),*s*<*k*.

### 3.3 Dynamic path analysis

The proposed approach can be viewed as a generalisation of dynamic path analysis,^7^, ^15^ which itself extends linear structural equation analysis to additive hazard models and normally distributed mediators, which obey additive linear models. To see this, we first consider a setting with a single mediator and no confounders for pedagogic purposes. Assuming an additive hazard model^21^ for the time‐to‐event outcome

$$\lambda (t|A,M_{1})=\lambda_{0}(t)+\lambda_{1}(t)A+\lambda_{2}(t)M_{1}I(t>1)$$

and a linear regression model for the mediator

$$E(M_{1}|T>1,A)=\alpha_{0}+\alpha_{1}A,$$

with normal errors and constant variance *σ* ^2^, we can evaluate expression (1) as follows. First, because the survival probability at time *t* is exp{−*H*(*t*)}, with *H*(*t*) the cumulative hazard at time *t*, the survival probability *P*(*T*>*t*|*T*>1,*A*,*M*_1_) can be evaluated as exp{−ΔΛ_0_(*t*)−ΔΛ_1_(*t*)*A*−ΔΛ_2_(*t*)*M*_1_} with ΔΛ_*j*_(*t*)=Λ_*j*_(*t*)−Λ_*j*_(1) and $\Lambda_{j}(t)=\int_{0}^{t}\lambda_{j}(s)ds$ for *j*=0,1,2 the cumulative hazard. Second, using that the moment generating function E{exp(Zt)} of a normal variate *Z*∼*N*(*μ*,*σ* ^2^) equals $\exp(\mu t+\frac{\sigma^{2}t^{2}}{2})$, integrating out *M*_1_ in expression (1) yields

$$\exp\left\{-\Delta \Lambda_{0}(t)-\Delta \Lambda_{1}(t)-\Delta \Lambda_{2}(t)E(M_{1}|T>1,A=0)-\frac{\Delta \Lambda_{2}{\left(t\right)}^{2}}{2}\mathrm{Var}(M_{1}|T>1,A=0)\right\},$$

which equals

$$\exp\left\{-\Delta \Lambda_{0}(t)-\Delta \Lambda_{1}(t)-\Delta \Lambda_{2}(t)\alpha_{0}-\frac{\Delta \Lambda_{2}{\left(t\right)}^{2}}{2}\sigma^{2}\right\}.$$

Finally, multiplying this result with *P*(*T*>1|*A*)=exp{−Λ_0_(1)−Λ_1_(1)*A*} yields

$$P(T_{1,0}>t)=\exp\left\{-\Lambda_{0}(t)-\Lambda_{1}(t)-\Delta \Lambda_{2}(t)\alpha_{0}-\frac{\Delta \Lambda_{2}{\left(t\right)}^{2}}{2}\sigma^{2}\right\}.$$

Likewise,

$$\begin{array}{ll} P(T_{0,0}>t) & =\exp\left\{-\Lambda_{0}(t)-\Delta \Lambda_{2}(t)\alpha_{0}-\frac{\Delta \Lambda_{2}{\left(t\right)}^{2}}{2}\sigma^{2}\right\}\\ P(T_{1,1}>t) & =\exp\left\{-\Lambda_{0}(t)-\Lambda_{1}(t)-\Delta \Lambda_{2}(t)\alpha_{0}-\Delta \Lambda_{2}(t)\alpha_{1}-\frac{\Delta \Lambda_{2}{\left(t\right)}^{2}}{2}\sigma^{2}\right\}, \end{array}$$

from which at each time *t*, the path‐specific effect not via *M* and via *M* can be calculated in terms of survival probabilities as

$$PDE(t)=\frac{S_{1,0}(t)}{S_{0,0}(t)}=\exp\{-\Lambda_{1}(t)\}$$

and

$$PIE(t)=\frac{S_{1,1}(t)}{S_{1,0}(t)}=\exp\left\{-\Delta \Lambda_{2}(t)\alpha_{1}\right\}=\exp\left\{-(\Lambda_{2}(t)-\Lambda_{2}(1))\alpha_{1}\right\},$$

respectively, for *t*>1 and 1 for *t* ≤ 1. These expressions hold more generally for studies with multiple waves if the event time obeys an additive hazard model at each time (conditional upon the information observed until that time) and the mediator in each wave of the study obeys a normal linear regression model with additive effects, constant variance, and normal errors. Similar expressions are also used in dynamic path analysis.^7^ The only difference lies in the indirect effect that has the additional term Λ_2_(1)×*α*_1_ in our proposal. This shows a first limitation of dynamic path analysis as currently considered in the work of Strohmaier et al^7^ in that this strategy ignores the potential for the event happening prior to the mediator assessment of the first wave. If all patients survive till the first mediator measurement, as in the examples in the work of Strohmaier et al,^7^ Λ_2_(1) equals zero and the approaches will coincide. As such, our results give formal justification for the expressions considered in dynamic path analysis as expressing path‐specific effects under the above listed assumptions. They also signal the other limitations of dynamic path analysis in that it is limited to specific additive models for the event time and the mediators, and that it cannot (easily) accommodate time‐varying confounders, nor lagged effects of the mediators.

## 4 EXAMPLE FROM THE LEADER TRIAL

LEADER was a multicentre, international, randomised, double‐blind clinical trial evaluating liraglutide (*A*=1) against placebo (*A*=0), both added to the standard of care. The trial was designed in accordance with guidance from FDA^22^ regarding the evaluation of cardiovascular risk for new antidiabetic therapies. In total, 9340 patients with type II diabetes at high risk for cardiovascular disease were randomised to one of the two treatment groups with a median follow‐up time of 3.8 years (ranging 3.5‐5.0 years). The subjects were attending planned visits 3 months after randomisation and subsequently every 6 months hereafter where at least HbA1c (*M*) was measured. The primary endpoint was the time *T* from randomisation to first occurrence of a so‐called major cardiovascular event (MACE) defined as nonfatal myocardial infarction, nonfatal stroke, or cardiovascular death. A first MACE occurred in significantly fewer patients in the liraglutide group (13.0%) than in the placebo group (14.9%) corresponding to an estimated hazard ratio of 0.87 (95% CI [0.78; 0.97]; P = 0.01) in the prespecified primary analysis, which was a Cox regression model with treatment as the only fixed effect.

Significant positive effects of liraglutide vs placebo were also found on cardiovascular risk factors such as glycated haemoglobin (HbA1c), body weight, urinary albumin to creatinine ratio, and blood pressure, and the interest is to evaluate to what extent these potential pathways might explain liraglutide's protective effect on cardiovascular events. The main results from the study can be found in the work of Marso et al.^1^

**Figure 2:**
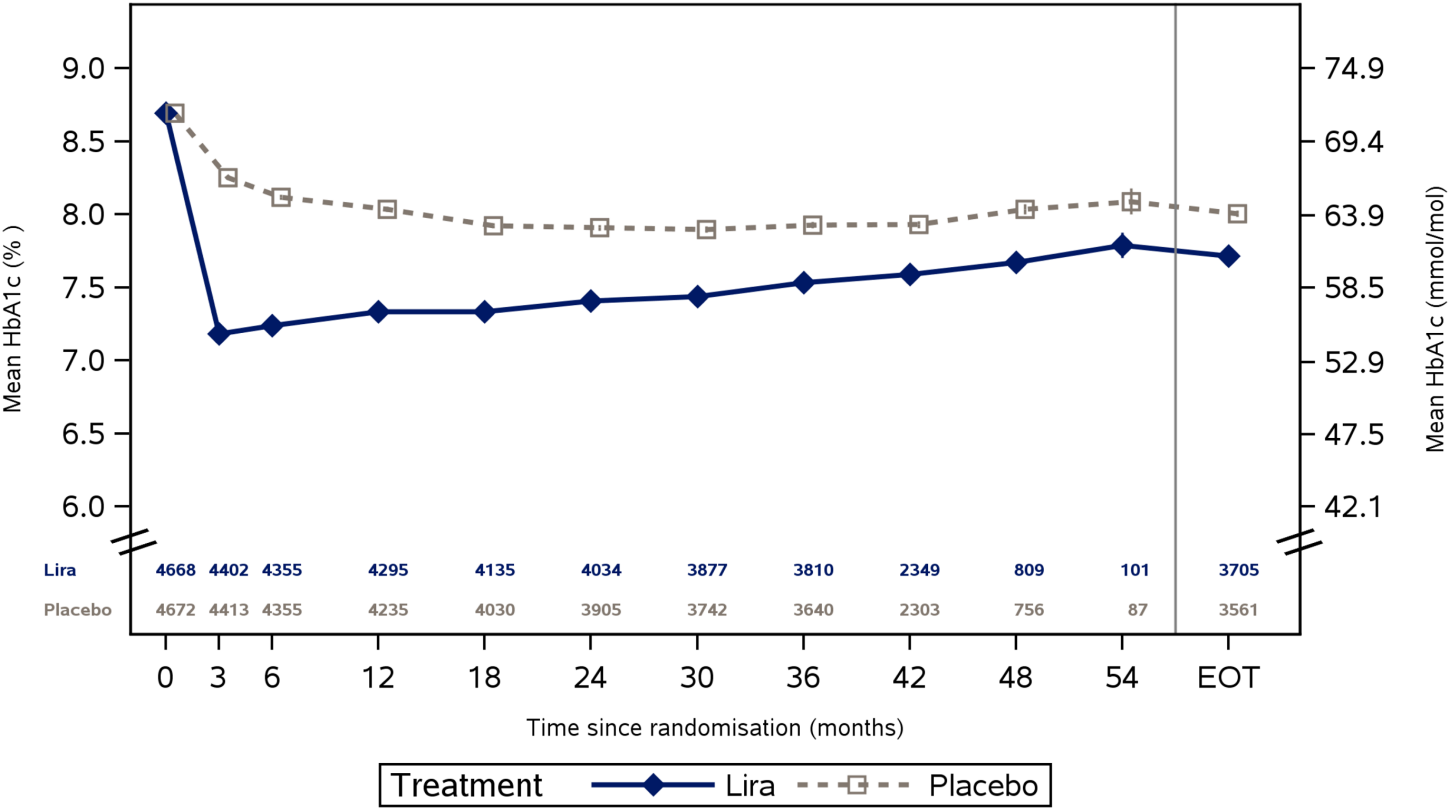
Estimated HbA1c levels over time by treatment group. EOT, end‐of‐trial visit (time varies by subject) [Colour figure can be viewed at http://wileyonlinelibrary.com]

**Figure 3:**
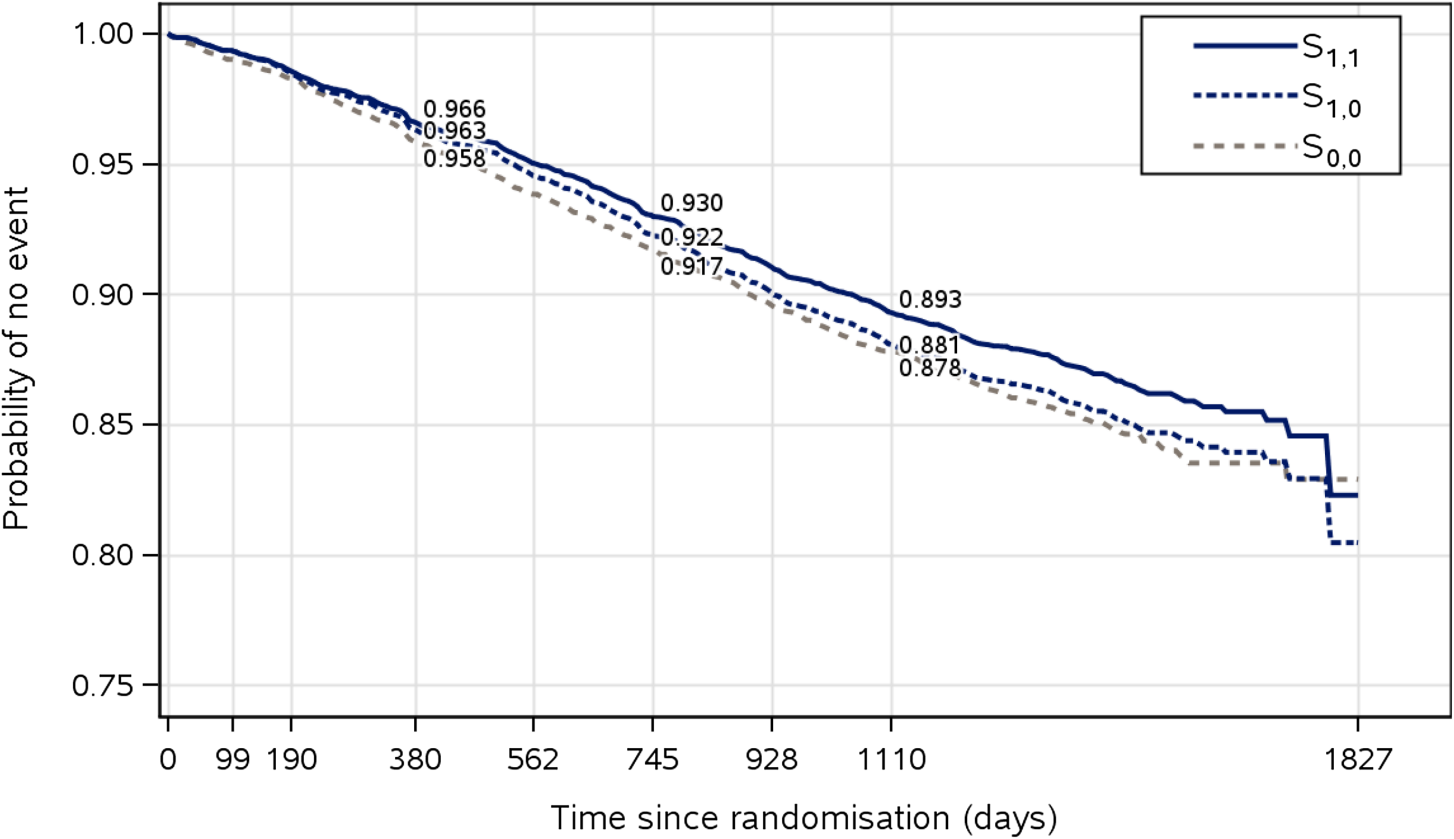
Estimated probabilities *S*_1,0_(*t*),*S*_1,1_(*t*), and *S*_0,0_(*t*) [Colour figure can be viewed at http://wileyonlinelibrary.com]

To illustrate the proposed approach for mediation analysis, we shall here restrict the attention to the potential mediation on the primary endpoint via the effect of liraglutide on HbA1c levels. Throughout, we will moreover assume that censoring is noninformative in the sense that the time to first MACE is equally distributed in patients who do versus those who do not discontinue the trial at a given time, but were assigned to the same arm and have the same history of HbA1c at that time. In Figure 2, the mean HbA1c levels over time are shown, as estimated by a mixed model for repeated measurements with adjustment for baseline covariates. As reported in the work of Marso et al,^1^ the estimated treatment difference was −0.40 percentage points (95% CI [−0.45; −0.34]) at the 36‐month visit, which was the last scheduled visit with laboratory testing for the entire trial population. In Figure 3, the estimated survival curves *S*_1,0_(*t*) are shown along with *S*_1,1_(*t*) and *S*_0,0_(*t*). The survival curves were estimated in accordance to the method presented in Section 3, where HbA1c measured after 3, 6, 12, 18, 24, 30, and 36 months, respectively, were included as mediators. Due to the novelty of the results, we could not present analyses that additionally adjusted for confounders other than supplementary HbA1c measurements, eg, body weight, and insulin use. Therefore, the results (which are also based on models with additive effects) are only to be considered hypothesis generating and should be interpreted with caution. The proposed method provides a framework for further analyses that can adjust for various confounders and explore other potential pathways than glycaemic control represented by HbA1c. More exhaustive analyses are reserved for subsequent communication in a medical journal.

**Figure 4:**
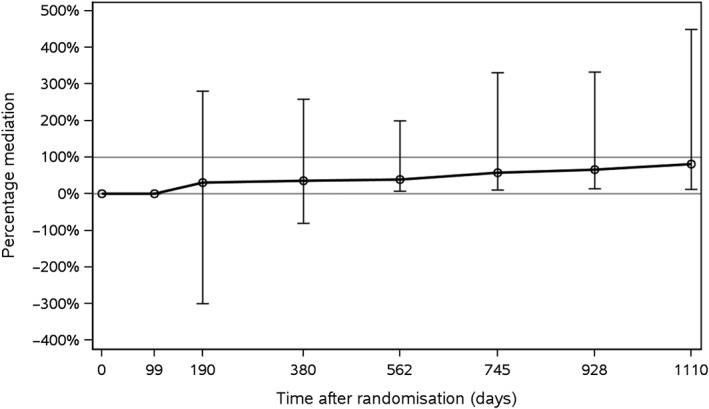
Mediated proportions for time to first major adverse cardiovascular event with longitudinal HbA1c levels as mediators

Figure 4 visualises the estimated mediated proportions at each visit. Inference was based on the nonparametric bootstrap with 1000 resamples. The results indicate that HbA1c is mediating parts of the effect of liraglutide on time to first MACE. Note however that the estimates of the mediated proportions are subject to uncertainty (reflected in the wide confidence intervals), and potentially residual confounding bias. Moreover, the mediated proportion appears to grow over time, whereas the total effect as measured by the hazard ratio is constant. This may suggest that the way by which glycaemic control represented by HbA1c biologically influences the cardiovascular risk may be complex. For instance, it may be the case that the mediated proportion over time depends on the number of available HbA1c measurements that are included in the analysis. Nearly identical results were found when body weight, urinary albumin to creatinine ratio, and systolic blood pressure were considered as additional potential mediators (not shown).

As a comparison to the method presented in Section 3, three relatively simple mediation analyses were also conducted:

1. Change in HbA1c after 6 months was used as mediator and included as a covariate in a Cox regression model with treatment as fixed factor, and baseline HbA1c as additional covariate.
2. Change in HbA1c over time was used as mediator and included as a time‐dependent covariate in a Cox regression model with treatment as fixed factor and baseline HbA1c as additional covariate.
3. Change in the trapezoidal area under the HbA1c curve divided by time (updated mean) was used as mediator and included as a time‐dependent covariate in a Cox regression model with treatment as fixed factor and baseline HbA1c as additional covariate.

For all three models, the mediated proportion was calculated as the difference in log hazard ratios between the models without and with the mediator, respectively, divided by the log hazard ratio from the model without the mediator. The results, which can be found in Table 2, are included because they represent rather standard analyses, even though they are known to be biased. Analyses 1 and 3 indicate some mediation. However, note that these mediated proportions have been calculated on different scales and are difficult to interpret because they reduce the mediator process to a single summary and, partly as a result, do not properly adjust for time‐varying confounding by the mediator history. As pointed out in Section 1, mediation analyses based on a single summary measure of the mediator may result in an underestimation of the mediated proportion, and this could very likely be the explanation for these differences. Furthermore, analysis 2 does not indicate any clear mediation, which is in line with the expectation that an analysis based on the last recorded value of the mediator will eliminate parts of an existing indirect effect. In addition, this analysis ignores confounding by the history of the mediators.

**Table 2:** Results from three simple mediation analyses

Model	Hazard ratio	95% CI	Mediated proportion
MACE primary analysis	0.87	(0.78; 0.97)	
Six‐month HbA1c change from the baseline	0.92	(0.81; 1.04)	0.40
as time‐fixed covariate			
HbA1c change from baseline	0.88	(0.79; 0.99)	0.08
as a time‐dependent covariate			
Updated mean of HbA1c	0.92	(0.82; 1.03)	0.40
as a time‐dependent covariate			

Abbreviation: MACE, major adverse cardiovascular event.

## 5 DISCUSSION

In this paper we have proposed a strategy to infer the effect of a randomised treatment on a time‐to‐event outcome as transmitted along the combination of pathways, whereby treatment directly—other than through a sufficient set of time‐varying confounders—influences one of a sequence of repeatedly measured mediator measurements *M*_*i*1_,…,*M*_*ik*_, which in turn influence the risk of the event of interest through an arbitrary mechanism. This proposal builds on the general theory on identification of path‐specific effects in nonparametric structural equations models with independent errors.^6^ It can be considered as a generalisation of the structural equation models extension to time‐to‐event outcomes, the so‐called dynamic path analysis,^7^ but can be used in more realistic settings as it can handle events happening prior to the first assessment of the mediator, is not limited to specific additive models for the event time and mediators, and easily accommodates time‐varying confounders and long‐term effects of mediators and covariates. As the general identification results in Web Appendix A are nonparametric, arbitrary models can be used in each of the steps of the procedure and our proposal is thus not limited to time‐to‐event outcomes. With a continuous outcome for instance, the Cox regression models at each wave would be replaced with a single model for the mean outcome at time *t*. Limited simulation studies in Web Appendix B demonstrate the adequate performance of the proposed methodology. SAS code for running this analysis is available in the online Supplementary Materials.

Our results shed light on the subtle interpretation of direct and mediated effects in studies with time‐to‐event endpoints. The notion of a direct effect conceptualises fixing the level of the mediator in the experimental treatment arm as it would have been on the control arm, but if a patient would have lived longer on the experimental treatment arm, then it becomes vague at what level the mediator ought to be controlled. For that reason, we have considered fixing the mediator at the level that would have been seen on the control arm if the patient had been kept alive for the same duration as in the experimental arm. In some cases, one may well hypothesise what would have happened if an event such as death had been prevented. For instance, if one of the study participants dies in a car crash, then we may well consider what that person's mediator level would have been at a given time had the car crash been prevented. In other cases, this is much harder to conceptualise. For instance, it is more difficult to imagine interventions that would prevent the event of interest in the considered patient populations. Strictly speaking one does not need to be precise about the kind of underlying interventions as inferences apply to all interventions that are noninvasive in the sense that if they had been applied to individuals who remained event free, the same data for the mediator would have been observed. However, it does complicate interpretation in the same way as treatment effects can be difficult to interpret in the presence of drop‐out due to death.^23^

A simple fix to the above problem can be made in extreme cases where treatment is beneficial for all patients. In that case, *S*_0,1_(*t*) is always well and unambiguously defined, and thus one can use the contrast of *S*_0,1_(*t*) and *S*_0,0_(*t*) as a measure of indirect effect, and the contrast of *S*_1,1_(*t*) and *S*_0,1_(*t*) as a measure of direct effect. Alternatively, and more generally, note that expression (1) can also be interpreted as a so‐called (randomised) interventional effect.^24^ In particular, as in the work of Zheng and van der Laan,^9^ it can be interpreted as the chance of being event free at time *t* if all patients were randomised to liraglutide and at each time *s*<*t*, the mediator were randomly drawn from the distribution of the mediator in patients in the control arm who survived time *s* and have the same history of covariate data (as observed under the considered regime). One potential concern here is that patients who survive time *s* on the control arm may fail to be comparable with those who survive time *s* on the intervention arm, even after adjusting for the history of time‐varying covariates. This may well happen as a result of survivor bias, due to which surviving patients may become more and more selective as time goes by. In our proposal, we have excluded this possibility by assuming the absence of unmeasured common causes of mediator (eg, *M*_1_) and time‐varying confounders (eg, *L*_1_) in the causal diagram of Figure 1. Such assumptions tend not to be spelled out in the existing approaches for (randomised) interventional direct and indirect effects. In addition, Didelez^10^ proposes related estimands. These estimands are well‐defined, even when patients on the control arm tend to experience events sooner than those on the intervention arm. They are moreover identifiable under weaker assumptions than the considered path‐specific effects, as they are not defined in terms of cross‐world counterfactuals, but have not been formally extended to settings with confounding by time‐varying covariates.

Our proposed approach is thus related to the works of Zheng and van der Laan^9^ and Didelez,^10^ but in contrast to these, focuses on the identification of path‐specific effects. VanderWeele and Tchetgen Tchetgen^18^ also adopted interventional direct and indirect effects, but they consider random draws from the distribution of the mediator at a certain exposure level conditional on only baseline covariate data. Their indirect effect, unlike ours, thus includes pathways whereby the treatment influences time‐varying confounders, then in turn influences the mediator and via that also the outcome. Their proposal also has the disadvantage that draws from the mediator distribution are ill defined when patients may die during the study. Lin et al^8^ handled this problem by redefining nested counterfactuals to include a counterfactual survival status in a similar way as in our proposal; in doing so, they eliminate pathways whereby treatment influenced the mediator via survival from the indirect effect, as in our proposal. However, in contrast to the work of Zheng and van der Laan^9^ and our proposal, they do not consider random draws from the mediator distribution conditional on time‐varying confounders. This raises questions whether the values drawn for a given patient will be sufficiently representative for what that patient might have “naturally” experienced, making these estimands less suitable to develop insight into mechanism. For instance, the assessment of a direct effect demands fixing the mediator at subject‐specific levels and it is unlikely that these can be “predicted” well when only baseline confounders are used for prediction.

Zheng and van der Laan^9^ deemed the assumptions needed to infer the path‐specific effects with multiple mediators and time‐varying confounders too strong for the purpose of effect mediation in a survival study. In Web Appendix A, we argue that following the arguments of Shpitser^6^ and the recanting witness criterion,^16^ the path‐specific effects represented by the causal diagram of Figure 1, are identified if (a) the exposure *A* is randomly assigned, (b) all common causes of the time‐to‐event outcome and the mediator at each time are measured, and (c) that this causal diagram represents a nonparametric structural equation model with independent error terms.^12^, ^25^ In particular, this means that the only variation in the variables (and their counterfactual values) on the causal diagram (not explained by previous variables in the diagram) is due to mutually independent error terms. Assumption (b) further implies that unmeasured common causes of the mediators over time are allowed (as long as they do not directly influence the time‐to‐event outcome), as well as unmeasured common causes of baseline and time‐varying confounders and the time‐to‐event endpoint. Note that common causes of time‐varying confounders and mediators are not allowed to be unmeasured.

The above results ignored competing risks due to death from noncardiovascular causes because the adjusted (Aalen‐Johansen) cumulative incidence curves were almost identical to the Kaplan‐Meier curves, suggesting that no appreciable differences can be expected when accounting for competing risks. However, the proposed approach can relatively easily be extended to handle competing risks. In that case, in each step of the algorithm, we substitute the probability of being event free by time *t* by the probability of being either event free by time *t* or having experienced a competing event. The latter can be calculated either by combining the results from standard Cox regression models for the two cause‐specific hazards for both causes,^26^ or using binomial regression models for the cumulative incidence. Another obvious extension to the above proposal is settings where the mediator is multivariate at each time, as may be the case when examining the effect mediated via, eg, glycated haemoglobin and body weight. It then infers the effect of treatment mediated via at least one of those mediators. This does not render the procedure any more complicated, in the sense that it requires no additional modelling. The above procedure also readily extends to enable decomposition of the treatment effects via multiple mediators. For instance, consider two mediators $M_{t}^{\left(1\right)}$ and $M_{t}^{\left(2\right)}$, and a vector of covariates $L_{t}^{*}$ at each time *t*=1,…,*k*, and suppose that $M_{t}^{\left(1\right)}$ may influence $M_{t}^{\left(2\right)}$, but not vice versa. Suppose furthermore that $L_{t}^{*}$ may influence $M_{t}^{\left(1\right)}$ and $M_{t}^{\left(2\right)}$, but not vice versa. Then, one may use the above procedure with $M_{t}\equiv M_{t}^{\left(2\right)}$ and $L_{t}\equiv (M_{t}^{\left(1\right)},L_{t}^{*})$ to infer the effect of treatment mediated via $M_{t}^{\left(2\right)}$ for *t*=1,…,*k*. One may likewise use the above procedure with $M_{t}\equiv M_{t}^{\left(1\right)}$ and $L_{t}\equiv (M_{t-1}^{\left(2\right)},L_{t}^{*})$ to infer the effect of treatment mediated via $M_{t}^{\left(1\right)}$ for *t*=1,…,*k*. When both effects are expressed on the risk difference scale, one may subtract the sum of both effects from the total effect *S*_1,1_(*t*)−*S*_0,0_(*t*) to obtain the direct treatment effect, which is not mediated by either $M_{t}^{\left(1\right)}$ or $M_{t}^{\left(2\right)}$, *t*=1,…,*k*.

## Supporting information

SIM_8336‐Supp‐0001‐Supplementary_Material.zip
