# Supplementary material for: Mediation analysis of time‐to‐event endpoints accounting for repeatedly measured mediators subject to time‐varying confounding
Source: Stat Med. 2019 Aug 14;38(24):4828–40. doi: 10.1002/sim.8336 (PMC6852414; doi:10.1002/sim.8336)
Supplement: Supplementary file 1 — SIM_8336‐Supp‐0001‐Supplementary_Material.zip [file SIM-38-4828-s001.zip › LongitudinalMediator-NovoNordisk-Vansteelandt_8_SM.pdf]

Mediation analysis of time-to-event endpoints accounting for repeatedly measured mediators subject to time-varying confounding: Supplementary Materials

Stijn Vansteelandt,
Ghent University, Belgium
and London School of Hygiene and Tropical Medicine, U.K.
stijn.vansteelandt@ugent.be
Martin Linder,
Novo Nordisk, Denmark
Sjouke Vandenberghe,
Ghent University, Belgium
Johan Steen,
Ghent University Hospital, Belgium
and Jesper Madsen
Novo Nordisk, Denmark

Web Appendix A: Identification and interpretation

In this Appendix, we demonstrate identification of the so-called path-specific effect via $M_1, M_2, ...$ under the assumptions embodied in the causal diagram of Figure **??**, where for simplicity we have dropped baseline covariates L_0 as well as measurements after the first two visits. All results given below generalize under a more general *complete* causal diagram (i.e. a causal diagram that includes all possible directed edges that follow the assumed temporal ordering of the observed variables) that may also include baseline

covariates L_0 and/or measurements for more than two study visits. In the diagram, L_1 represents time-varying confounders measured at visit 1, along with the indicator of having survived the first visit of the study. That is, $L_1 = (I(T > 1), I(T > 1)V_1)$; this is needed because V_1 is ill-defined if $T < 1$. Let L_2 represent time-varying confounders measured at visit 2, including the indicator of having survived the second visit of the study: $L_2 = (I(T > 2), I(T > 2)V_2)$ The key assumptions sufficient for identification:

- that the exposure A is (conditionally) randomized (given exposure history or baseline covariates L_0).

- that at each time t, a set of covariates is available that is sufficient to adjust for confounding on the effect of M_t on the time-to-event endpoint T; however, note that common causes of the mediators M over time are allowed to be unmeasured, as well as common causes of confounders L and time-to-event endpoint T.

- that the causal diagram represents a nonparametric structural equation model with independent errors (1).

- the consistency assumptions that $L_1(a) = L_1$ for individuals with exposure $A = a$, that $M_1(a, l_1) = M_1$ for individuals with exposure $A = a$ and covariate $L_1 = l_1$, ... and the composition assumptions that $M_1(a, L_1(a)) = M_1$, $L_2(a, L_1(a), M_1(a, L_1(a))) = L_2$, ...

Allowing for the above structure of unmeasured confounding, the set of observed variables in the associated causal diagram generally partitions into 3 *districts* (2) or *c-components* (3) (e.g. $\{A\}$, $\{M_1, M_2\}$ and $\{L_1, L_2, T\}$ in Figure **??**). Because the path-specific effect π on which we focus (i.e. the natural indirect effect via M_1 and M_2) consists of the combination of all paths that feed directly from A to either M_1 or M_2, continuing

to T along any remaining pathway (i.e. the set of pathways $\pi = \{A \to M_1 \to T, A \to M_2 \to T, A \to M_1 \to M_2 \to T, A \to M_1 \to L_2 \to T, A \to M_1 \to L_2 \to M_2 \to T\}$), none of the districts is recanting with respect to π (4). In other words, there does not exist a directed path in π of the form $A \to D \to ... \to T$ as well as a directed path not in π of the form $A \to D' \to ... \to Y$, where both D and D' belong to a common district (and possibly $D = D'$).[1] If there would exist such a path, then the district to which D and D' belong, would be called a recanting district with respect to π and the π-specific effect would not be identifiable.

This is immediately seen because all pathways leaving A that are included in π directly intercept the district $\{M_1, M_2\}$. It follows from (4) that this path-specific effect is therefore identifiable under a NPSEM representation of the causal diagram. In particular, for any $2 < t \leq 3$, the counterfactual probability

$$\begin{aligned} S_{a,a^*}(t) &= P\{T(a, L_1(a), M_1(a^*, L_1(a)), L_2(a, M_1(a^*, L_1(a))), M_2(a^*, L_1(a), \\ &\quad M_1(a^*, L_1(a)), L_2(a, M_1(a^*, L_1(a)))) > t\} \qquad (1) \end{aligned}$$

is identifiable as

$$\int f\{T(a, m_1, m_2) > t, L_1(a) = l_1, L_2(a, m_1) = l_2\} \times f\{M_1(a^*, L_1) = m_1, M_2(a^*, l_1, l_2) = m_2\}\, dl_1 dm_1 dl_2 dm_2$$

It further follows from (3) that $f\{T(a, m_1, m_2) > t, L_1(a) = l_1, L_2(a, m_1) = l_2\}$ equals

$$\begin{aligned} & P(T > t | A = a, l_1, m_1, l_2, m_2) f(L_2 = l_2 | A = a, l_1, m_1) f(L_1 = l_1 | A = a) \\ & = P(T > t | T > 2, A = a, l_1, m_1, l_2, m_2) f(V_2 = v_2 | T > 2, A = a, l_1, m_1) \\ & \quad \times P(T > 2 | T > 1, A = a, l_1, m_1) f(V_1 = v_1 | T > 1, A = a) P(T > 1 | A = a) \end{aligned}$$

[1]Strictly speaking, one needs to consider districts within a particular subgraph of the original causal diagram (4). However, when considering *complete* causal diagrams (such as Figure **??**) with the aforementioned structure of unmeasured confounding, the set of districts in the associated subgraph corresponds to those in the original causal diagram with the exception of the district $\{A\}$.

and $f\left\{M_1(a^*,l_1)=m_1, M_2(a^*,l_1,l_2)=m_2\right\}$ equals

$$f(M_1=m_1|T>1,A=a^*,l_1)f(M_2=m_2|T>2,A=a^*,l_1,m_1,l_2).$$

In the presence of baseline covariates L_0, all terms in the above expressions require additional conditioning on L_0.

For a general t, one can find likewise that

$$\begin{aligned} S_{a,a^*}(t) &= \int P\left(T>t|T>\lfloor t\rfloor, \overline{m}_{\lfloor t\rfloor}, \bar{l}_{\lfloor t\rfloor}, A=a,\right) \prod_{s=1}^{\lfloor t\rfloor} f\left(m_s|T>s,\bar{l}_s,\overline{m}_{s-1},A=a^*\right) \\ & \times f\left(v_s|T>s,\bar{l}_{s-1},\overline{m}_{s-1},A=a\right) P\left(T\geq s|T>s-1,\bar{l}_{s-1},\overline{m}_{s-1},A=a\right) dm_s dl_s, \end{aligned}$$

where we define $\overline{m}_s \equiv (m_1,...,m_s)$,$\bar{l}_s \equiv (l_1,...,l_s)$, $m_0=\emptyset$ and $l_0=\emptyset$. Again, in the presence of baseline covariates L_0, all terms in the above expressions require additional conditioning on L_0, and the averaging is also over the distribution of L_0. Furthermore, identification of the terms $P\left(T\geq s|T>s-1,\bar{l}_{s-1},\overline{m}_{s-1},A=a\right)$ and $P\left(T>t|T>\lfloor t\rfloor,\overline{m}_{\lfloor t\rfloor},\bar{l}_{\lfloor t\rfloor},A=a,\right)$ requires non-informative censoring assumptions stated in the main text below the detailed Monte Carlo algorithm.

In section 3.3 in the main text, we propose a general estimation procedure based on repeated regressions. For instance, for $1<t\leq 2$ this procedure builds on the following result

$$\begin{aligned} S_{1,0}(t) &\overset{step1}{=} \int \underbrace{P(T>t|T>\lfloor t\rfloor, A=1, \overline{m}_{\lfloor t\rfloor}, \bar{l}_{\lfloor t\rfloor})}_{Q^{\lfloor t\rfloor}(t)} \prod_{s=1}^{\lfloor t\rfloor} f(m_s|T>s,A=0,\overline{m}_{s-1},\bar{l}_s) \\ & \quad \times f(l_s|T>s-1,A=1,\overline{m}_{s-1},\bar{l}_{s-1}) dm_s dl_s \\ &= \int Q^{\lfloor t\rfloor}(t) f(m_{\lfloor t\rfloor}|T>\lfloor t\rfloor, A=0, \overline{m}_{\lfloor t\rfloor-1}, \bar{l}_{\lfloor t\rfloor}) f(l_{\lfloor t\rfloor}|T>\lfloor t\rfloor-1, A=1, \overline{m}_{\lfloor t\rfloor-1}, \bar{l}_{\lfloor t\rfloor-1}) \\ & \quad \times \prod_{s=1}^{\lfloor t\rfloor-1} f(m_s|T>s,A=0,\overline{m}_{s-1},\bar{l}_s) f(l_s|T>s-1,A=1,\overline{m}_{s-1},\bar{l}_{s-1}) dm_{\lfloor t\rfloor} \\ & \quad dm_s dl_{\lfloor t\rfloor} dl_s \end{aligned}$$

$$
\begin{aligned}
&\overset{step2(a)}{=} \int \underbrace{E[Q^{\lfloor t \rfloor}(t)|T > \lfloor t \rfloor, A=0, \overline{m}_{\lfloor t \rfloor -1}, \bar{l}_{\lfloor t \rfloor}]}_{Q_m^{\lfloor t \rfloor}(t)} f(l_{\lfloor t \rfloor}|T > \lfloor t \rfloor -1, A=1, \overline{m}_{\lfloor t \rfloor -1}, \bar{l}_{\lfloor t \rfloor -1}) \\
&\qquad \times \prod_{s=1}^{\lfloor t \rfloor -1} f(m_s|T > s, A=0, \overline{m}_{s-1}, \bar{l}_s) f(l_s|T > s-1, A=1, \overline{m}_{s-1}, \bar{l}_{s-1}) dm_s dl_{\lfloor t \rfloor} dl_s \\
&= \int \underbrace{E[Q^{\lfloor t \rfloor}(t)|T > \lfloor t \rfloor, A=0, \overline{m}_{\lfloor t \rfloor -1}, \bar{l}_{\lfloor t \rfloor}]}_{Q_m^{\lfloor t \rfloor}(t)} f(v_{\lfloor t \rfloor}|T > \lfloor t \rfloor, A=1, \overline{m}_{\lfloor t \rfloor -1}, \bar{l}_{\lfloor t \rfloor -1}) \\
&\qquad \times P(T > \lfloor t \rfloor | T > \lfloor t \rfloor -1, A=1, \overline{m}_{\lfloor t \rfloor -1}, \bar{l}_{\lfloor t \rfloor -1}) \\
&\qquad \times \prod_{s=1}^{\lfloor t \rfloor -1} f(m_s|T > s, A=0, \overline{m}_{s-1}, \bar{l}_s) f(l_s|T > s-1, A=1, \overline{m}_{s-1}, \bar{l}_{s-1}) dm_s dv_{\lfloor t \rfloor} dl_s \\
&\overset{step2(b)}{=} \int \underbrace{E[Q_m^{\lfloor t \rfloor}(t)|T > \lfloor t \rfloor, A=1, \overline{m}_{\lfloor t \rfloor -1}, \bar{l}_{\lfloor t \rfloor -1}]}_{Q_l^{\lfloor t \rfloor}(t)} P(T > \lfloor t \rfloor | T > \lfloor t \rfloor -1, A=1, \overline{m}_{\lfloor t \rfloor -1}, \bar{l}_{\lfloor t \rfloor -1}) \\
&\qquad \times \prod_{s=1}^{\lfloor t \rfloor -1} f(m_s|T > s, A=0, \overline{m}_{s-1}, \bar{l}_s) f(l_s|T > s-1, A=1, \overline{m}_{s-1}, \bar{l}_{s-1}) dm_s dl_s \\
&\overset{step2(c)}{=} \int \underbrace{Q_l^{\lfloor t \rfloor}(t) P(T > \lfloor t \rfloor | T > \lfloor t \rfloor -1, A=1, \overline{m}_{\lfloor t \rfloor -1}, \bar{l}_{\lfloor t \rfloor -1})}_{Q^{\lfloor t \rfloor -1}(t)} \\
&\qquad \times \prod_{s=1}^{\lfloor t \rfloor -1} f(m_s|T > s, A=0, \overline{m}_{s-1}, \bar{l}_s) f(l_s|T > s-1, A=1, \overline{m}_{s-1}, \bar{l}_{s-1}) dm_s dl_s \\
&= \int Q^{\lfloor t \rfloor -1}(t) \prod_{s=1}^{\lfloor t \rfloor -1} f(m_s|T > s, A=0, \overline{m}_{s-1}, \bar{l}_s) f(l_s|T > s-1, A=1, \overline{m}_{s-1}, \bar{l}_{s-1}) dm_s dl_s \\
&\overset{step2}{=} \ldots = \int Q^1(t) f(m_1|T > 1, A=0, \bar{l}_1) f(l_1|A=1, l_0) f(l_0) dl_0 dl_1 \\
&= \int Q^0(t) f(l_0) dl_0 \\
&\overset{step3}{=} E\left\{Q^0(t)\right\}
\end{aligned}
$$

The indices above the equality signs refer to the corresponding steps described in the general procedure in the main paper.

The path-specific effects inferred in the article are based on contrasts, such as (1), of nested counterfactuals corresponding to the same value of a, but different values of a^*.

Expression (1) clarifies their interpretation, considering the problem that, for instance, $M_1(0)$ is ill-defined for subjects who would have died prior to wave 1 if they were assigned to placebo (5). For instance, let $L_t = I(T > t)$ for $t = 1, 2$ for simplicity of notation. Then, for $t > 2$, (1) expresses how likely it would be to survive time t, if (a) one were assigned to treatment level a, and (b) the mediator at time 1 were set to the value it would have taken if one were assigned to treatment a^* and kept alive until wave 1 or the time to death under treatment regimen a, whichever comes first, and (c) the mediator at time 2 were set to the value it would have taken if one were assigned to treatment a^* and kept alive until wave 2 or the time to death under treatment regimen a, whichever comes first (see steps a and b).

Web Appendix B: Simulations

To evaluate the behavior of our proposal, a simulation study was performed with 1000 runs for data sets of 1000 observations. We first describe the data-generating strategy for the exposure, time-varying covariates, mediators and the survival outcome. Next results for three simulation scenarios are presented: no direct effect, no indirect effect because the exposure has no effect on the mediators and the mediators have no effect on the outcome, and no indirect effect only because the exposure has no effect on the mediators. We focus on these 3 settings because there is often much interest in testing for the presence of 'direct' or 'indirect' effects, and moreover because the magnitude of the true effects is known in these settings.

To generate data, first, a dichotomous exposure A is drawn with $P(A = 0) = P(A = 1) = 0.5$. We assumed a study period of 5 years with visits at $0.5, 2$ and 4 years at which the mediators $M_{0.5}, M_2$ and M_4 and time-varying covariates $L_{0.5}, L_2$ and L_4 were assessed. To simulate covariates $L_0, L_{0.5}, L_2$ and L_4, we first generated a random intercept and a

random time slope for all 1000 observations via a multivariate normal distribution with mean intercept and slope (μ_{l0}, μ_{l1}) equal to $(65, 0.3)$ and covariance matrix with variances $(\sigma^2_{\mu_{l0}}, \sigma^2_{\mu_{l1}})$ equal to $(15, 0.1)$ and correlation -0.2. Similarly, to simulate mediators $M_{0.5}, M_2$ and M_4, we first generated a random intercept and a random time slope for all 1000 observations via a multivariate normal distribution with mean intercept and slope (μ_{m0}, μ_{m1}) equal to $(4, -0.2)$ and covariance matrix with variances $(\sigma^2_{\mu_{m0}}, \sigma^2_{\mu_{m1}})$ equal to $(1, 0.1)$ and correlation -0.3.

Next $L_0, L_{0.5}, L_2$ and L_4 are drawn from a normal distribution with variance 1 and for L_0 mean equal to $\mathtt{E}(L_0) = \mu_{l0i}$, for $L_{0.5}$ equal to $\mathtt{E}(L_{0.5}|t, A) = \mu_{l0i} + \mu_{l1i}t + \alpha_{l1}A$, for L_2 equal to $\mathtt{E}(L_2|t, A, M_{0.5}) = \mu_{l0i} + \mu_{l1i}t + \alpha_{l2}A + 0.1M_{0.5}$ and for L_4 equal to $\mathtt{E}(L_4|t, A, M_2) = \mu_{l0i} + \mu_{l1i}t + \alpha_{l3}A + 0.05M_2$, with $(\alpha_{l1}, \alpha_{l2}, \alpha_{l3})$ equal to $(0, 0, 0)$ in the no direct effect setting and equal to $(0.5, 1, 1.2)$ in the no indirect effect settings. $M_{0.5}, M_2$ and M_4 are also drawn from a normal distribution with variance 1 and for $M_{0.5}$ mean equal to $\mathtt{E}(M_{0.5}|t, A, L_{0.5}) = \mu_{m0i} + \mu_{m1i}t + \alpha_{m1}A + 0.05L_{0.5}$, for M_2 equal to $\mathtt{E}(M_2|t, A, L_2) = \mu_{m0i} + \mu_{m1i}t + \alpha_{m2}A + 0.075L_2$ and for M_4 equal to $\mathtt{E}(M_4|t, A, L_4) = \mu_{m0i} + \mu_{m1i}t + \alpha_{m3}A + 0.1L_4$, with $(\alpha_{m1}, \alpha_{m2}, \alpha_{m3})$ equal to $(-2, -1.75, -1.5)$ in the no direct effect setting and equal to $(0, 0, 0)$ in the no indirect effect settings.

Finally, a time-to-event outcome T is drawn at each visit (i.e. T_0 before $L_{0.5}$ and $M_{0.5}$ are known, $T_{0.5}$ before L_2 and M_2 are known $\ldots$) from a Weibull distribution with shape parameter $a = 1$ and scale parameter $b = 1/\{\lambda_T(t)\exp(0.01\mu_{l0i} + \beta_{l0}L_0 + \beta_{at}A)\}$ for $t < 0.5$ and $b = 1/\{\lambda_T(t)\exp(0.01\mu_{l0i} + \beta_{l0}L_0 + \beta_{at}A + \beta_{lt}L_t + \beta_{mt}M_t)\}$ for $t \geq 0.5$. Once the simulated event time was smaller than the time of the next visit, we assumed that the event took place for that patient and the event time was saved. For patients with generated event times larger than the time of the next visit, a new event time is generated in the next wave and added to the known time this patient would have survived (i.e. the

time of the previous visit). This process was repeated for all visits, after the final visit event times were saved for all remaining patients. Patients with event times larger than 5 years were censored. To generate a time-varying baseline hazard $\lambda_T(t)$ we applied cubic splines and specified the function to take the values $(0.1, 0.2)$ at years $(0, 5)$. Further β_{at} took values $(0, 0, 0, 0)$ to generate $T_0, T_{0.5}, T_2$ and T_4 in the no direct effect setting and $(-1, -1, -1, -1)$ in the no indirect effect settings. β_{lt} took values $(-0.01, -0.02, -0.04)$ to generate $T_{0.5}, T_2$ and T_4 in the no direct effect setting, $(0.01, 0.02, 0.04)$ in the first no indirect setting and $(0.005, 0.01, 0.02)$ in the second no indirect effect setting. β_{mt} took values $(0.15, 0.2, 0.225)$ to generate $T_{0.5}, T_2$ and T_4 in the no direct effect setting, $(0, 0, 0)$ in the first no indirect effect setting and $(0.1, 0.15, 0.2)$ in the second no indirect effect setting. Finally β_{l0} took the value -0.01 in the no direct effect setting and -0.03 in the no indirect effect settings. Note that μ_{l0} and μ_{m0} respectively serve as U_l and U_m as in Figure **??**.

Results of the simulation analyses for the three settings are presented in Figures 1, 2 and 3 respectively. Results show that in all settings, as predicted by the theory, the non-parametric total effect estimate equals the total effect as estimated via the proposal in Section **??**. Additionally, there is indeed no evidence of a direct or indirect effect in the first setting and the second and third setting respectively.

Figures 1, 2 and 3 about here.

References

[1] VanderWeele TJ, Vansteelandt S, Robins JM. Effect decomposition in the presence of an exposure-induced mediator-outcome confounder.. *Epidemiology* 2014; 25(2): 300-306.

[2] Richardson TS. A factorization criterion for acyclic directed mixed graphs, In: Proceedings of the Twenty-Fifth Conference on Uncertainty in Artificial Intelligence. In: 2009 (pp. 462-470).

[3] Tian J, Pearl J. A General Identification Condition for Causal Effects, In: Proceedings of the Eighteenth National Conference on Artificial Intelligence (AAAI). In: Menlo Park, CA: AAAI Press/The MIT Press. 2002 (pp. 567-573).

[4] Shpitser I. Counterfactual graphical models for longitudinal mediation analysis with unobserved confounding.. *Cognitive Science* 2013; 37(6): 1011-1035.

[5] Lin SH, Young JG, Logan R, VanderWeele TJ. Mediation analysis for a survival outcome with time-varying exposures, mediators, and confounders. *Statistics in medicine* 2017; 36(26): 4153–4166.

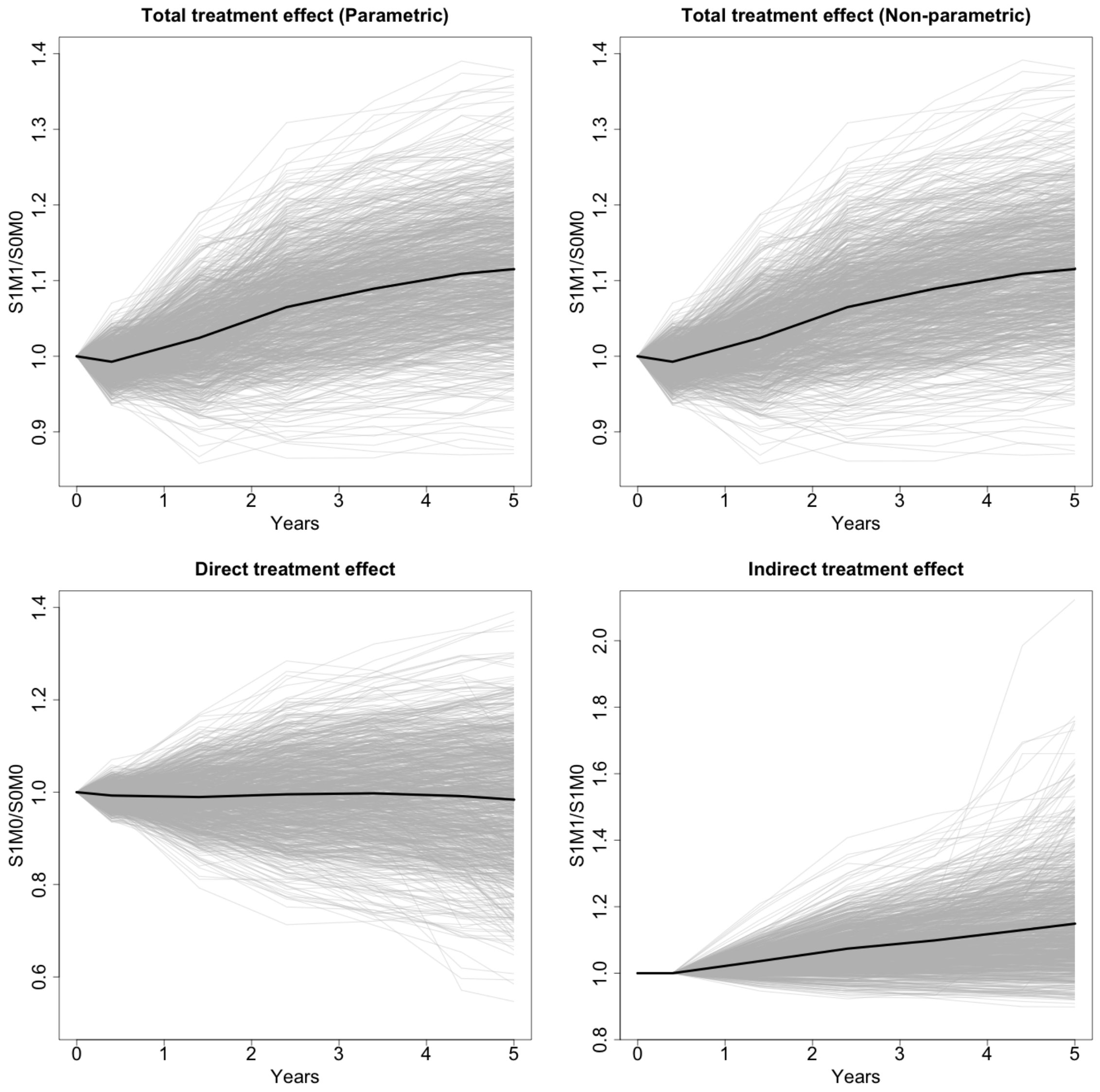

Figure 1: Total (parametric and non-parametric), direct and indirect effect in the no direct effect setting

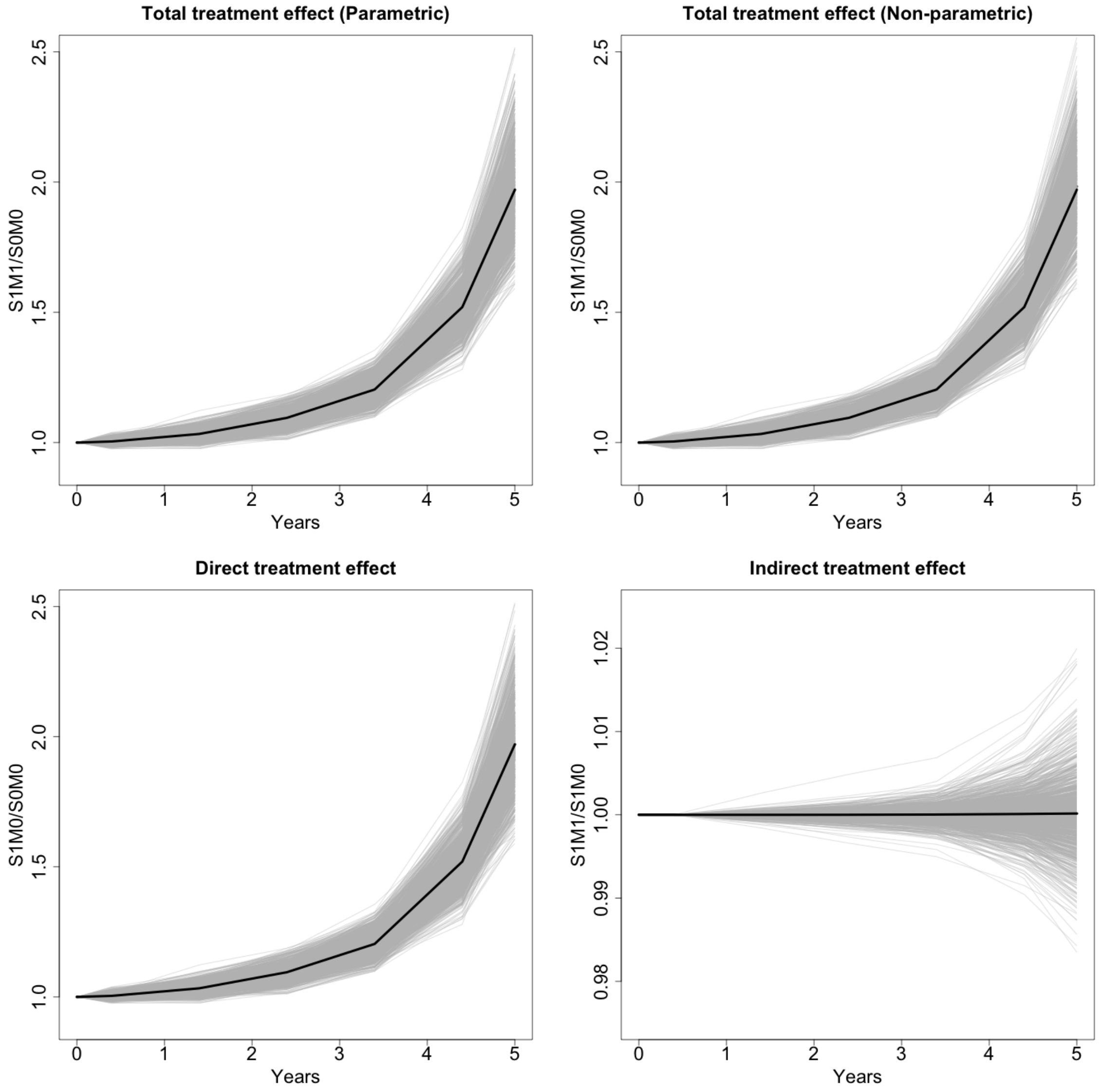

Figure 2: Total (parametric and non-parametric), direct and indirect effect in the first no indirect effect setting

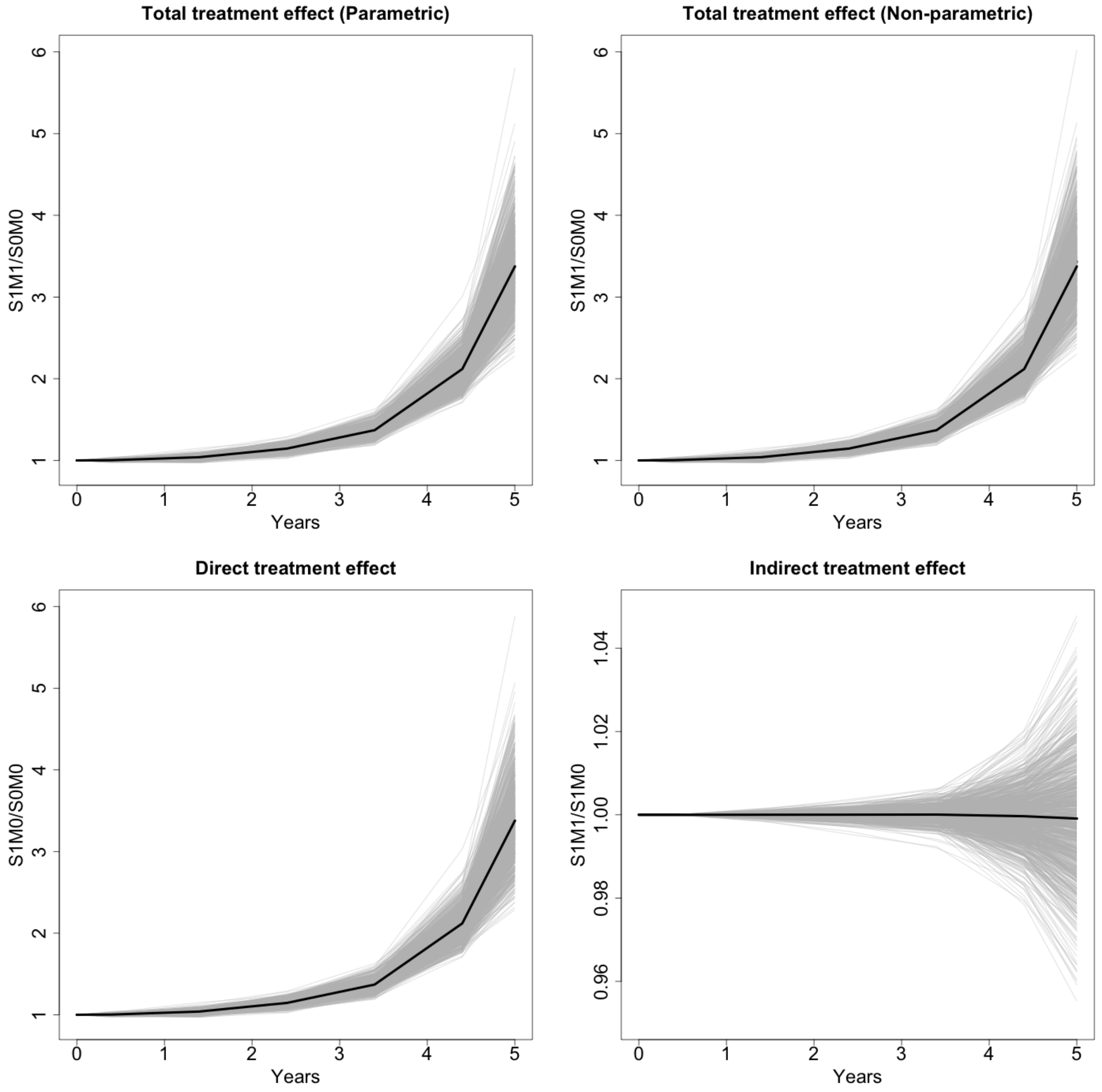

Figure 3: Total (parametric and non-parametric), direct and indirect effect in the second no indirect effect setting
